# Incarcerated Interparietal Hernia at an 8-mm Robotic Port Site after Robot-Assisted Left Lateral Sectionectomy despite External Oblique Fascial Closure: A Case Report

**DOI:** 10.70352/scrj.cr.26-0713

**Published:** 2026-11-10

**Authors:** Yuzuru Sakamoto, Tatsuhiko Kakisaka, Akihisa Nagatsu, Shunsuke Shichi, Sunao Fujiyoshi, Takeshi Aiyama, Norio Kawamura, Masaaki Watanabe, Ryoichi Goto, Akinobu Taketomi

**Affiliations:** Department of Gastroenterological Surgery I, Hokkaido University Graduate School of Medicine, Sapporo, Hokkaido, Japan

**Keywords:** robot-assisted hepatectomy, port-site hernia, trocar-site hernia, interparietal hernia, small-bowel obstruction

## Abstract

**INTRODUCTION:**

Port-site hernia at an 8-mm robotic trocar site is uncommon, and routine fascial closure of these sites is not currently regarded as mandatory. In the interparietal variant, bowel herniates through defects in the peritoneum, transversalis fascia, transversus abdominis, and internal oblique layer while remaining beneath an intact external oblique layer, producing little or no abdominal wall bulge.

**CASE PRESENTATION:**

An 80-year-old woman underwent robot-assisted left lateral sectionectomy for hepatic alveolar echinococcosis using the da Vinci Xi system. She had undergone 4 previous abdominal operations, and extensive adhesiolysis was required; the operative time was 291 min. At each of the 3 standalone 8-mm robotic port sites, the fascia of the external oblique muscle was identified through the skin incision under direct vision and closed with a single suture, and no drain was placed through a port site. Abdominal radiographs on PODs 1 and 4 showed no signs of bowel obstruction, but on POD 5, the patient vomited. No abdominal wall bulge was appreciable, but CT showed a small-bowel loop incarcerated at the left flank 8-mm port site on the anterior axillary line, lying between the external and internal oblique muscles. Emergency laparoscopic surgery was performed on the same day, and the previous fascial suture was found to be intact. The bowel was reduced from the intermuscular plane and remained viable, so resection was avoided; omentum had also entered the right flank 8-mm port site and was reduced. Both defects were then closed full-thickness, including the peritoneum, under laparoscopic vision. She was discharged 10 days later and remained well at 4 months.

**CONCLUSIONS:**

An interparietal port-site hernia can occur at a lateral 8-mm robotic port despite closure of the external oblique fascia and may present without a visible or palpable bulge. Prompt CT is warranted when early postoperative obstructive symptoms develop. Superficial closure alone may be insufficient in some situations, but whether full-thickness closure, including the peritoneum, prevents this complication remains to be established.

## INTRODUCTION

The da Vinci platform uses a characteristic 8-mm trocar, and these sites are often left unclosed. This practice reflects the very low incidence reported in the available studies: in a prospective study of 166 patients and 513 unclosed 8-mm trocar sites, only 1 asymptomatic hernia was detected, and no patient became symptomatic.^1)^ A literature review covering 18533 patients reported an overall trocar-site hernia rate of about 0.1% and found no significant difference between closed and unclosed 5 and 10-mm sites.^2)^ The current European and American Hernia Societies guideline recommends aponeurotic closure for trocar sites of 10 mm or larger, albeit as a weak recommendation based on very low-quality evidence.^3)^ No specific recommendation has been established for the 8-mm robotic port.

Although uncommon, hernias at these sites appear to present acutely relatively often. In a retrospective series of 433 8-mm robotic ports, the hernia rate was 0.7%, and 2 of the 3 affected patients required emergency repair for small-bowel incarceration in the early postoperative period.^4)^ In a review of 11566 robotic abdominal operations, acute port-site hernia occurred in 15 patients (0.13%). Eleven of the 15 acute port-site hernias (73%) occurred at 8-mm robotic port sites, 2 of which required small-bowel resection.^5)^

Diagnosis may be further complicated when herniation is confined between the muscle layers. In the interparietal variant, also termed intramuscular or intrafascial, bowel passes through defects in the peritoneum, transversalis fascia, transversus abdominis, and internal oblique layer but remains beneath an intact external oblique layer, so little or no bulge is apparent.^6,7)^ At 8-mm robotic trocar sites, this pattern has been described only after urological procedures,^6)^ and it has recently been reported at a 12-mm robotic port after colorectal surgery.^8)^

We report an incarcerated interparietal hernia at a lateral 8-mm robotic port site after robot-assisted left lateral sectionectomy, occurring in a patient in whom the fascia of the external oblique muscle at that site had been sutured at the index operation and was found to be intact at reoperation. To our knowledge, this combination of a lateral 8-mm robotic port, an interparietal pattern of herniation, and an intact superficial closure has not been reported previously; we are also not aware of a previous report of an 8-mm robotic port-site hernia after robot-assisted liver resection.

## CASE PRESENTATION

An 80-year-old woman was found to have a hepatic lesion in the left lateral section during a routine health screening examination. Serological testing for *Echinococcus multilocularis* was positive by Western blot, and CT showed a cystic lesion without contrast enhancement. Hepatic alveolar echinococcosis was diagnosed, and the lesion was classified as P1N0M0, stage I. Her medical history included hypertension, dyslipidemia, and paroxysmal atrial fibrillation. She had undergone 4 previous abdominal operations: appendectomy, cholecystectomy for cholelithiasis, total hysterectomy for uterine myoma, and adhesiolysis for small-bowel obstruction. She was not receiving anticoagulant or antiplatelet therapy.

She was 138.1 cm tall and weighed 57 kg, giving her a BMI of 29.9 kg/m^2^. Liver function was Child–Pugh class A, with an indocyanine green retention rate at 15 min of 10.9%; the complete blood count, biochemistry, and coagulation studies were unremarkable. As the lesion was confined to the left lateral section, left lateral sectionectomy was planned, and a robot-assisted approach was selected.

### Index operation

Robot-assisted left lateral sectionectomy was performed with the da Vinci Xi surgical system (Intuitive Surgical, Sunnyvale, CA, USA). Port placement is shown in **Fig. 1**. A wound retractor with an EZ Access platform (Hakko, Nagano, Japan) was mounted at the umbilicus and carried the 8-mm camera port (arm 2), together with a 5-mm port prepared for the Pringle maneuver. Three standalone 8-mm robotic ports were placed: arm 1 in the right flank, arm 4 in the left flank on the anterior axillary line approximately 3 cm cranial to the umbilicus, and arm 3 between the umbilicus and the arm 4 port. A 12-mm assistant port was placed between the umbilicus and the arm 1 port. All robotic ports were the 8-mm blunt-tipped (non-bladed) trocars supplied with the system, and the assistant port was a 12-mm Endopath Xcel bladeless trocar (Ethicon, Johnson & Johnson, Cincinnati, OH, USA). Extensive adhesions from previous surgery required prolonged adhesiolysis. Parenchymal transection was performed with dual bipolar forceps, and the Pringle maneuver was not used. The operative time was 291 min with minimal blood loss, and there were no intraoperative adverse events. The resected left lateral section was retrieved through the umbilical wound.

**Fig. 1:**
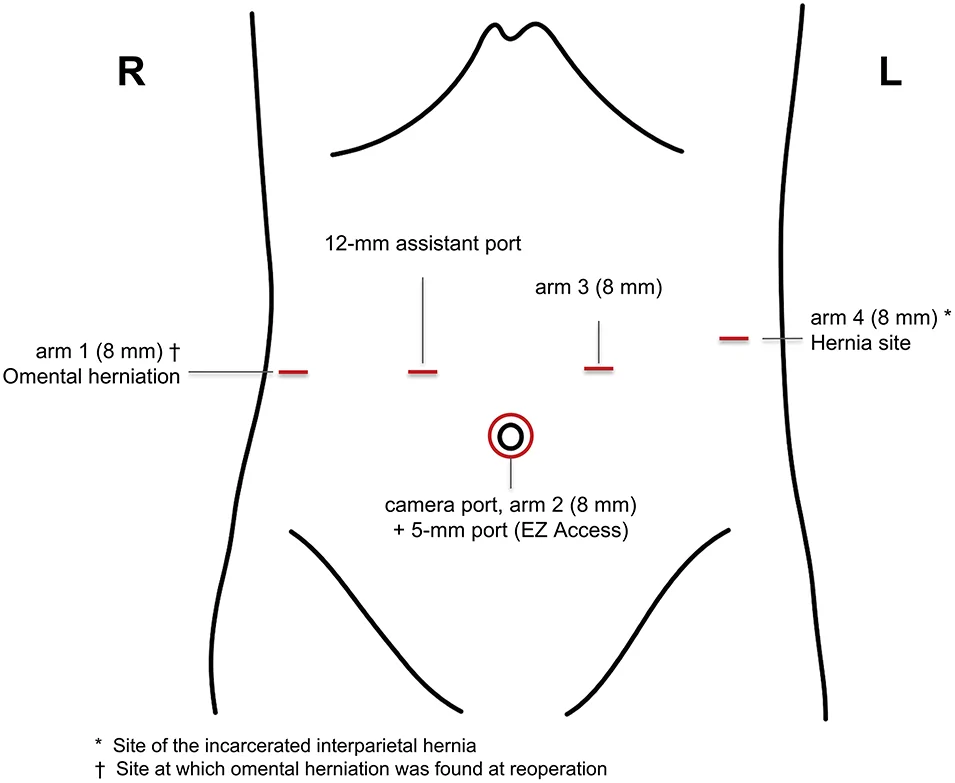
Port placement at the index robot-assisted left lateral sectionectomy. A wound retractor with an EZ Access platform at the umbilicus carried the 8-mm camera port (arm 2), together with a 5-mm port prepared for the Pringle maneuver. Three standalone 8-mm robotic ports were placed: arm 1 in the right flank, arm 4 in the left flank on the anterior axillary line approximately 3 cm cranial to the umbilicus, and arm 3 between the umbilicus and the arm 4 port. A 12-mm assistant port was placed between the umbilicus and the arm 1 port. The asterisk indicates the arm 4 site at which the incarcerated interparietal hernia developed, and the dagger indicates the arm 1 site at which omental herniation was found at reoperation. L, patient’s left; R, patient’s right

At closure, the umbilical wound was closed with interrupted 0 polydioxanone (PDS Plus; Ethicon) sutures under direct vision, and the fascia at the 12-mm assistant port site was closed with a Z-suture of 2-0 polyglactin 910 under direct vision. At each of the 3 standalone 8-mm robotic port sites, closure was performed through the original 8-mm skin incision without extension: the incision was retracted with small retractors, the fascia of the external oblique muscle was identified under direct vision and grasped with a Kocher clamp, and a single 2-0 polyglactin 910 suture (Vicryl; Ethicon) was placed. At the level of the arm 4 site, the trocar traversed the muscular portion of the external oblique, so the layer closed was the fascia investing the muscle rather than its aponeurosis. The same technique was used at the arm 1 and arm 3 sites. The internal oblique and transversus abdominis layers and the peritoneum were not closed, and closure was not confirmed under laparoscopic vision from within the abdominal cavity. No drain was placed through any port site. Histopathological examination confirmed hepatic alveolar echinococcosis, and the resection was R0.

### Postoperative course and reoperation

Oral intake was resumed on POD 1, and the early course was satisfactory. Plain abdominal radiographs obtained on POD 1 and POD 4 showed no small-bowel gas or air-fluid levels. On POD 5, the patient vomited. Laboratory tests showed no notable abnormality, and no appreciable bulge was noted at any port site on inspection or palpation.

Contrast-enhanced CT demonstrated a loop of small bowel incarcerated at the left flank 8-mm port site (robotic arm 4), with proximal small-bowel dilatation and a collapsed distal segment (**Fig. 2A**–**2C**). The herniated loop lay not in the subcutaneous plane but between the external and internal oblique muscles, with the external oblique muscle and its fascia being continuous over it. There were no findings suggestive of strangulation, such as pneumatosis intestinalis, mesenteric fat stranding, or ascites. Manual reduction was attempted but was unsuccessful, and emergency surgery was undertaken on the same day.

**Fig. 2:**
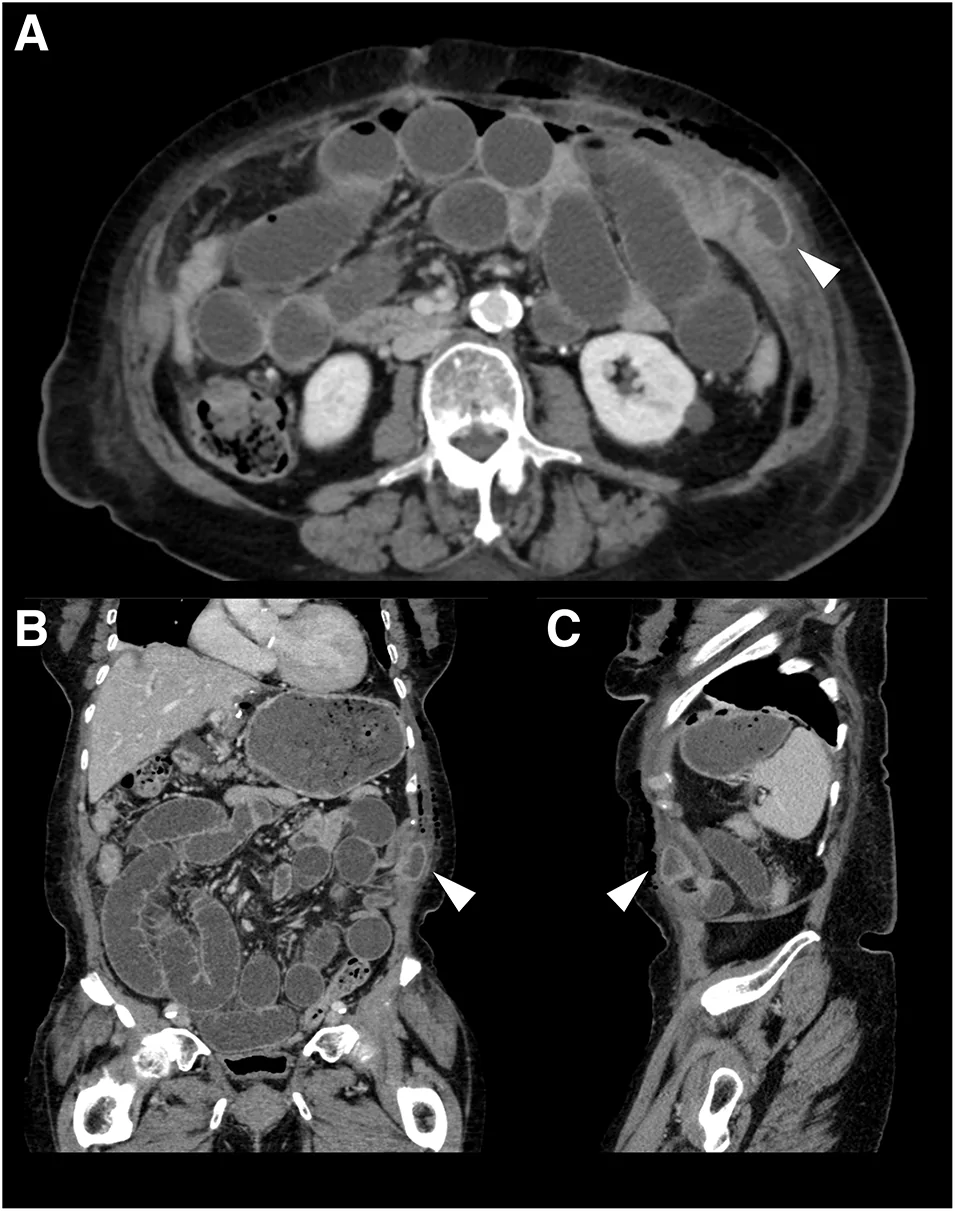
Contrast-enhanced CT on POD 5. Axial (**A**), coronal (**B**), and sagittal (**C**) images show a loop of small bowel incarcerated at the left flank 8-mm port site (arrowhead), lying between the external and internal oblique muscles beneath the intact external oblique muscle and fascia, with dilatation of the proximal small bowel.

Under general anesthesia, a wound retractor with an EZ Access platform was mounted on the umbilical extraction wound used at the index operation, and one 12-mm port and two 5-mm ports were introduced through it. The small bowel was seen entering the deep defect at the left flank 8-mm port site and tracking into the plane between the internal and external oblique muscles (**Fig. 3A**). The anatomical configuration is illustrated in **Fig. 3C**. The loop was drawn back into the peritoneal cavity without difficulty, after which the fascial defect at the arm 4 site was clearly seen from the peritoneal side (**Fig. 3B**). The skin incision at this site was reopened, and palpation confirmed that the previous suture in the external oblique fascia was intact. Inspection of the remaining port sites showed that omentum had also entered the right flank 8-mm port site (robotic arm 1), and this was likewise reduced. This omental herniation had not been identified on CT; laparoscopically, the omentum had been drawn beyond the peritoneum into the port tract. The arm 1 site had been closed in the same manner as the arm 4 site; its skin incision was likewise reopened, and palpation confirmed that the fascial suture was intact. The omentum therefore appeared to have remained beneath the external oblique fascia, although the depth to which it had reached could not be determined with certainty. Immediately after reduction, the serosa was erythematous, but its appearance improved with observation. Perfusion was well preserved, and there was no necrosis or perforation; bowel resection was therefore not performed. Both the left and right flank defects were then closed full-thickness, including the peritoneum, under direct laparoscopic vision using a suture-passer device (Endo Close; Medtronic, Minneapolis, MN, USA). The operative time was 84 min, and the blood loss was minimal.

**Fig. 3:**
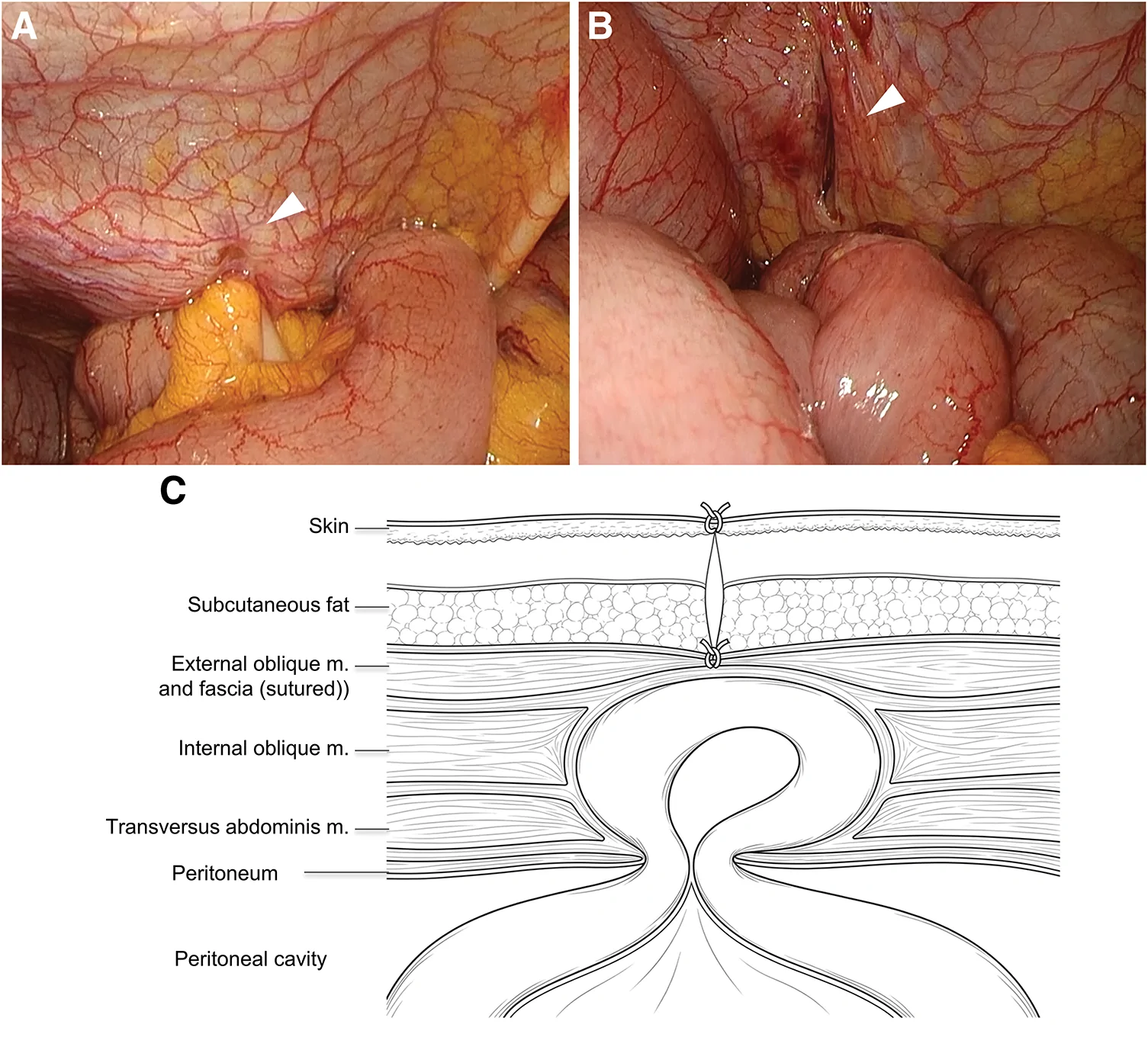
Intraoperative findings at reoperation and the anatomy of the hernia. (**A**) Intraoperative laparoscopic view at the reoperation showing the small bowel entering the deep defect at the 8-mm port site (arrowhead). (**B**) The fascial defect at the arm 4 site after reduction of the herniated bowel (arrowhead). (**C**) Schematic representation of the interparietal hernia: the bowel passes through defects in the peritoneum, transversalis fascia, transversus abdominis, and internal oblique layer and lies beneath the sutured external oblique fascia.

The subsequent course was uneventful. Oral intake was resumed on the 3rd day after the 2nd operation, and the patient was discharged 10 days after reoperation. She remained well without recurrent obstruction or abdominal wall bulge at 4 months after discharge.

### Change in institutional practice

After this case, we changed our closure policy for 8-mm robotic port sites on the lateral abdominal wall. These sites are now closed full-thickness, including the peritoneum, under laparoscopic vision at the end of the procedure using a suture-passer device, rather than being closed at the level of the external oblique fascia from the skin side.

## DISCUSSION

Trocar-site hernias are classically associated with midline and umbilical ports of 10 mm or larger, and 8-mm robotic ports are commonly left unclosed on the basis of prospective and comparative data showing very low hernia rates.^1–3)^ Nevertheless, the small number of reported 8-mm hernias tend to present early and with incarceration. Damani et al. found that 11 of 15 acute postoperative robotic port-site hernias (73%) arose at 8-mm sites and that 2 of these 11 patients required small-bowel resection.^5)^ Published cases have presented between POD 2 and POD 9, in several instances with bowel necrosis requiring resection.^4,6,9–11)^ Our patient presented on POD 5; CT was obtained promptly, surgery was performed the same day, and the bowel remained viable.

Within general surgery, 8-mm port-site hernias have been reported after robotic cholecystectomy^12)^ and after robotic ileocecal resection,^13)^ but in both cases, the affected sites lay in the lower abdomen. We are not aware of a previous report after robot-assisted hepatectomy. However, the main point of the present case lies less in the index operation than in the combination of a lateral 8-mm robotic port, an interparietal pattern of herniation, and a superficial closure that was confirmed to be intact at reoperation.

In the present case, the fascia of the external oblique muscle at the affected site had been sutured under direct vision and remained intact, yet herniation occurred beneath it. Furuke et al. recently reported an intramuscular port-site hernia after robot-assisted colorectal surgery despite closure of the external oblique aponeurosis; however, their case involved a 12-mm port and required small-bowel resection.^8)^ The present case differs in that incarceration occurred through an 8-mm robotic port after liver resection, and omental herniation was also identified at a 2nd 8-mm port site.

Closure limited to the outermost layer may not address the level at which herniation begins. A trocar creates a defect through the peritoneum, transversalis fascia, transversus abdominis, and internal oblique layer, as well as the external oblique layer, and it is the deep opening that admits bowel. When only the external oblique layer is closed through a small skin incision, the deeper layers cannot be seen even if the superficial layer is identified under direct vision, and they may remain open. Lim et al., reporting an interparietal hernia at an 8-mm robotic trocar site after robot-assisted laparoscopic prostatectomy, likewise emphasized that 8-mm sites associated with a large peritoneal defect warrant careful closure.^6)^ In a report of an 8-mm port-site hernia after robot-assisted radical prostatectomy, the authors noted that secure closure of the fascia and peritoneum had not been confirmed from within the abdominal cavity.^11)^

An intact outer layer also limits the abdominal wall bulge that would otherwise be apparent. Chiong et al. described 4 intrafascial hernias among 1055 laparoscopic urological procedures, in which bowel protruded through defects in the transversalis fascia and internal oblique layer while the external oblique aponeurosis was preserved. They noted that such hernias are difficult to appreciate on physical examination and that CT is important for diagnosis.^7)^ Our patient similarly had no appreciable swelling despite an incarcerated loop, and the presenting feature was vomiting alone.

Several factors may have contributed, but they are supported by evidence of different strength. Among patient-related factors, obesity has been associated with trocar-site hernia in a clinical series,^14)^ and the patient’s BMI was 29.9 kg/m^2^. Advanced age and multiple previous abdominal operations were also present and have been noted in a comparable case report,^9)^ although their independent contributions have not been established. The operative time was prolonged (291 min) because of extensive adhesiolysis. Of note, no drain had been placed through the affected port site, although drain placement through an 8-mm port is a recognized risk factor and accounted for more than a 3rd of acute hernias in 1 series.^5)^

By contrast, the following mechanisms are speculative. Repeated instrument exchange and sustained torque at the trocar during prolonged adhesiolysis, or displacement of the remote center, may enlarge the fascial aperture,^11)^ but the size of the defect at the end of the index operation was not measured. The lateral abdominal wall comprises 3 laminae that a trocar traverses obliquely, so defects in successive layers may not be superimposed, which could favor an interparietal course. Diez-Barroso et al. suggested that sliding of the overlapping muscle layers after release of pneumoperitoneum occludes the trocar tract and that this effect is weaker where the trocar passes through aponeurosis or tendon.^4)^ Because the arm 4 trocar in the present case traversed the muscular portion of the external oblique, this mechanism alone does not explain the hernia. Reports of 8-mm hernias at anterior axillary line and left lateral port sites^10,15)^ nonetheless suggest that lateral port sites warrant attention. Finally, the presence of omental herniation at a 2nd 8-mm site suggests that patient-level abdominal-wall factors, rather than conditions unique to the affected port alone, may also have contributed.

Whether all 8-mm robotic port sites should be routinely closed remains uncertain because the absolute incidence is very low. A recent meta-analysis found that fascial closure reduced the relative risk of trocar-site hernia, but the absolute risk difference was small and not statistically significant.^16)^ Closure also adds operative time and carries a theoretical risk of nerve or muscle injury at the lateral abdominal wall. What this single case suggests is more limited: closing only the superficial layer from the skin side may be insufficient to prevent deep-layer herniation in some situations. Full-thickness closure including the peritoneum under laparoscopic vision is 1 option, and we have adopted it for lateral 8-mm port sites; however, a single case cannot demonstrate that it prevents port-site hernia, and its benefit would need to be evaluated in larger series.

Regardless of closure policy, new abdominal pain, vomiting, or ileus after robotic surgery merits prompt CT, and the absence of a palpable bulge does not exclude a port-site hernia.

## CONCLUSIONS

An interparietal port-site hernia can occur at a lateral 8-mm robotic port despite closure of the external oblique fascia and may present without a visible or palpable bulge. Prompt CT is warranted when early postoperative obstructive symptoms develop. Superficial closure alone may be insufficient in some situations, but whether full-thickness closure including the peritoneum prevents this complication remains to be established.

## DECLARATIONS

### Availability of data and materials

All relevant data supporting this case report are included in the article. Additional clinical data are not publicly available to protect patient privacy.
